# Face-to-Face or Telematic Cognitive Stimulation in Patients with Multiple Sclerosis and Cognitive Impairment: Why Not Both?

**DOI:** 10.1155/2017/5713934

**Published:** 2017-12-13

**Authors:** C. Guijarro-Castro, Y. Aladro-Benito, A. Sánchez-Musulim, A. Belen-Caminero, I. Pérez Molina, I. Gómez-Moreno, L. Gómez-Romero, J. Millán-Pascual, M. J. Laredo, M. Cerezo-García

**Affiliations:** ^1^Sección de Neurología, Hospital Virgen de la Luz, Cuenca, Spain; ^2^Sección de Neurología, Hospital Universitario de Getafe de Madrid, Madrid, Spain; ^3^Hospital Santa Bárbara de Puertollano, Ciudad Real, Spain; ^4^Sección de Neurología, Hospital Ntra. Sra. de Sonsoles de Ávila, Ávila, Spain; ^5^Hospital Universitario Virgen de la Salud de Toledo, Toledo, Spain; ^6^Sección de Neurología, Hospital La Mancha Centro de Alcazar de San Juan, Ciudad Real, Spain

## Abstract

**Introduction:**

Cognitive impairment (CI) affects 40–65% of patients with multiple sclerosis (MS). Few studies address telematic cognitive stimulation (TCS) in MS. The objective of this study is to evaluate the efficacy and impact of telestimulation or distance cognitive stimulation (TCS), with and without the support of face-to-face cognitive stimulation (FCS) in cognitive impairment in MS.

**Methods:**

Multicentre, prospective, randomised, controlled study. We will include 98 MS patients with EDSS ≤ 6, symbol digit modality test (SDMT) ≤ Pc 25, and Multiple Sclerosis Neuropsychological Screening Questionnaire (MSNQ) > 26 points. Patients will be randomised into 3 groups, a TCS group, a mixed TCS/FCS group, and a control group. CS is performed 3 days a week for 3 months. Processing speed, memory, attention, and executive functions will be rehabilitated. FCS will include ecological exercises and strategies. EDSS and a cognitive evaluation (SDMT, CTMT, PASAT, and TAVEC), MSNQ, psychological impact scales (MSIS), and depression (BDI) will be carried out, baseline, postrehabilitation, and also 6 and 12 months later, to evaluate the effect of CS in the longer term.

**Conclusion:**

This study could help to establish the usefulness of TCS or, in its absence, TCS with face-to-face help for CI in MS. The interest lies in the clear benefits of remote rehabilitation in the daily life of patients.

## 1. Introduction

Multiple sclerosis (MS), a chronic demyelinating disease, is characterised by a heterogeneous set of symptoms that can lead to severe disability and have an impact on accessibility to medical services, functional capacity of the patient, and health-related quality of life (HRQOL) [1].

Cognitive impairment (CI) affects 40–65% of patients and mainly involves information processing speed (IPS), attention, executive functions, and memory. It becomes more frequent as the disease evolves, but may appear in early stages and be independent of physical disability. IC correlates mainly with cerebral atrophy estimated by magnetic resonance (MR) volumetric techniques [2–4]. In MS, deficits in concentration, attention, working memory, IPS, and executive functions, predominantly in problem solving and abstract reasoning [5], are more frequently observed. Deficits vary from series to series in relation to, among other factors, the neuropsychological batteries used and the type of patient studied. Two neuropsychological batteries have been widely accepted for the study of CI in MS, the Rao Brief Battery (BRB-N) [6], and the MACFIMS (Minimal Assessment of Cognitive Function in Multiple Sclerosis) [7]. Both have the inconvenience of administration time, 30 and 90 minutes, respectively, and require qualified personnel. The Symbol Digit Modalities Test (SDMT) (about 2 minutes for execution) has been proposed as a screening test because of its high sensitivity and specificity to discriminate patients with and without CI. It is a good tool in longitudinal studies because of its reproducibility [8]. Recently, the BICAMS (Brief International Cognitive Assessment of Multiple Sclerosis) has been proposed as a new screening battery. This has been validated in 28 countries and supported by different neurological associations. It takes 15 minutes, can be performed by nonspecialised staff, and includes the California Verbal Learning Test-II (CVLT-II), the Brief Visuospatial Memory Test-Revised, and the SDMT [9].

There is no pharmacological treatment for CI in MS [10]. There is a sufficient consensus on the protective role of intellectual enrichment in the development of CI in dementia and aging. In MS, the work is with lower numbers but the results are similar: level of education and the intellectual enrichment maintained by leisure activities such as reading, among others, diminish the deleterious effect of the lesional load and atrophy in cognition [11, 12]. There are few studies that analyse the effect of cognitive rehabilitation on MS's cognitive dysfunction [13]. Recent analyses by Cochrane [14, 15] show that intensive training, specifically in memory, seems to have a clear benefit. Fifteen phase III studies with a total of 989 participants are included, but the risk of bias is low in only 7 of these and heterogeneity is important. The results are more contradictory with attention; there is little work with rehabilitation programmes aimed at treating IPS and executive functions [8, 9, 14, 15]. Functional MRI studies have shown that this CS activates the prefrontal and cingulate cortexes [16].

### 1.1. Justification and Hypotheses

No comparative studies comparing telematic (TCS) and face-to-face stimulation (FCS) with neuropsychologists are currently known. Telestimulation would offer clear advantages for the patient, given its lower interruption in their daily activities, work, social life, and so forth. With the design of this randomised and controlled pilot study, we want to compare individualised training by TCS with face-to-face support, with individualised training only by TCS.

### 1.2. Objectives of the Study

#### 1.2.1. Main Goal

The main goal is to evaluate the efficacy and impact of TCS with and without FCS support on CI.

#### 1.2.2. Secondary Objectives

1. Evaluate the degree of patient satisfaction with the TCS programme.
2. Evaluate adherence to treatment with the TCS programmes.
3. Evaluate if the changes obtained with the rehabilitation treatment are maintained at 6 and 12 months of the CS.

## 2. Material and Methods

This is an experimental, prospective, randomised, controlled, minimally interventional, and multicentre study.

The study is being carried out in the following hospitals, with the approval of the Research Ethics Committee of each centre: Hospital Virgen de la Luz (Cuenca), Hospital Universitario Virgen de la Salud (Toledo), Hospital Santa Bárbara (Ciudad Real), Hospital Virgen de Sonsoles (Ávila), Hospital Valdepeñas (Ciudad Real), and Hospital Universitario de Getafe (Madrid).

Patients with MS according to the McDonald Criteria, 2010 (1917) from multiple sclerosis consultations ranging from 18–65 years old, EDSS (Expanded Disability Status Scale, 1983) ≤6, with a score of >26 in the Multiple Sclerosis Neuropsychological Screening Questionnaire (MSNQ) [17] and/or ≤Pc 25 in the SDMT [18], have signed informed consent.

Exclusion criteria are severe cognitive impairment (defined by the Mini-Mental State Examination (MMSE) scores ≤ 26) [19], being free of attacks and/or treatment with corticosteroids 30 days before the first cognitive assessment, presence of other CNS disease, history of vascular or traumatic brain damage, and psychiatric disorder or psychotropic substance abuse that may interfere with the test performance.

The patients included will be randomised into 3 groups: (1) TCS group, patients will receive only telematic intervention for 3 days each week; (2) mixed TCS/FCS group, patients will receive telematic intervention for 2 days more FCS for one day each week; and (3) control group. The face-to-face rehabilitation will be performed with 2 neuropsychologists in groups of five patients. As in the design of another recent clinical trial on cognitive rehabilitation, our control group will be assigned to the waiting list, providing the opportunity to participate in the cognitive rehabilitation programme once the study has been completed [20].

### 2.1. Neurological and Neuropsychological Evaluation

Patients from all three groups will undergo a neuropsychological evaluation that includes the domains of IPS, attention, memory, and executive functions, before starting CS (baseline visit), at the end of 12 weeks of treatment (visit 2) and at 6 and 12 months.

The neurological and neuropsychological evaluation is detailed as follows:

1. Disability: EDSS, baseline, and after training
2. Neuropsychological testing
  - Processing speed: SDMT [18], Comprehensive Trail Making Test (CTMT) CTMT 1 [21]
  - Attention: CTMT 2 y 3 [20]
  - Executive functions: PASAT 3 (Paced Auditory Serial Addition Test) [22], CTMT 4 and 5, verbal fluency (phonological and semantic)
  - Memory: Spain-Complutense Verbal Learning Test (TAVEC) (Spanish version of the California Verbal Learning Test) [23]
3. Self-administered questionnaires
  - Risk for neuropsychological impairment and self-perceived cognitive quality of life: MSNQ [17]
  - Fatigue: Fatigue Severity Scale (FSS) and Fatigue Impact Scale (FIS) [24]
  - Physical and psychosocial impact of MS: Multiple Sclerosis Impact Scale (MSIS-29) [25]
  - Depression: Beck Depression Inventory (BDI) [26]
  - Satisfaction measure: satisfaction questionnaire

#### 2.1.1. Cognitive Stimulation Programme

*(1) Cognitive Telestimulation Programme*. A state-of-the-art cognitive stimulation application will be installed on the latest generation devices (computers, tablets, and smartphones). This platform is based on a web resource and mobile applications and uses games, validated [27, 28] and selected by expert neuropsychologists. This computer programme has been validated in fibromyalgia, healthy students and children with attention deficit hyperactivity disorder [27, 28]. It has different training settings (“full brain,” “athletes,” “students,” “drivers,” “executives,” “over 60,” and “children”) and allows users to obtain a profile of the advances obtained by cognitive stimulation. In addition, they are sent reminder messages to perform assigned tasks. They have 62 games classified according to the predominant cognitive domain, “perception and speed,” “memory,” “attention,” and “executive functions.”

At each training session, patients will perform 4 sets (one from each cognitive domain), with an estimated time of 15 minutes for all four games.

*(2) Cognitive Stimulation Face-to-Face in Cognitive Strategies*. It will be based on exercises and teach strategies with ecological exercises based on principles of optimisation, compensation, and restoration, to improve cognition (one session), attention (2 sessions), memory (2 sessions), and executive functions (one session on inhibition and another on problem solving). There will be 3 more sessions for presentation and adherence to the programme, feedback on the CS software, and evaluation of the programme.

### 2.2. Statistical Analysis

We have calculated the sample size with an online platform (http://Fisterra.com). The sample size estimate is based on an analysis of the MSIS. Starting from a population of 450 patients with MS, a sample size of 98 patients with CI and MS representative of MSIS has been calculated.

The SPSS.22.0 program will be used for statistical analysis.

The primary outcome was a cognitive performance measured by improvement in SDMT, PASAT, CTMT and TAVEC, and self-perceived psychosocial impact (MSNQ, MSIS-29).

Continuous variables will be expressed as mean and standard deviation or median and 25th and 75th percentiles and categorical variables as percentages. The normal distribution of the data will be verified by the Kolmogorov–Smirnov test. To compare baseline demographic, clinical, and neuropsychological variables between groups, one-way ANOVA or Kruskal-Wallis will be used for quantitative variables and Chi-square or Fisher's exact test for categorical variables.

The effect of CS on cognitive performance was measured in each group (named “intragroup”) comparing cognitive scores before and after treatment using an ANCOVA (analysis of covariance) adjusted by depression level or Friedman test for independent samples. To compare outcomes in cognitive scores between groups, an ANCOVA adjusted by sex, age, education level, baseline cognitive scores, and depression level will be used. The same procedure will be employed to analyse the effect of CS on MSIS and MSNQ as daily life impact measures.

Patients will be stratified in 2 groups: cognitive improvement and no changes/impairment in order to evaluate if age, sex, EDSS, educational level, baseline cognitive status, and FCS support (independent variables) have a capacity of predicting the cognitive improvement (dependent variable) through a logistic regression model. Cognitive improvement is defined as a gain > 1.5 S.D. in the score in at least 2 cognitive tests.

This study has been awarded a grant for research in the XIII Call for Research Grants of 2016 from the Mutua Madrileña Foundation.

## 3. Discussion

Telemedicine (TM) is the exchange of medical information between two different physical locations. The goals of TM are to provide services that cannot be managed face-to-face and improve the efficiency of existing ones. The use of TM to clinically monitor MS patients has been shown to improve Health-related quality of life (HRQOL), reducing the cost of associated medical services [29]. A randomised clinical trial has shown that cognitive behavioural therapy was an effective treatment for fatigue in MS, using an internet-based version programme consisting of eight interactive sessions with clinical psychologists [30]; although studies with a greater number of patients are needed.

A recent Spanish study demonstrated the usefulness of TM to assess gait disability in MS patients, especially in the most disabled. The gait distance evaluation included a video of self-performed neurological examination and specific multimedia questionnaires, together with the measurements of a triaxial accelerometer [31].

Several studies about impact of cognitive rehabilitation in MS, although with methodology limitations [32], show some improvements in attention, IPS, executive functions, and working memory [33, 34]. They have few patients and are not comparable and thus do not confirm the effectiveness reported. Cochrane has performed a systematic review on TCS in MS patients, including studies using telecommunication technology in their environment [35]. They included nine randomised clinical trials (*N* = 531 participants, 469 patients included) investigating a variety of TCS interventions in adults with MS. These interventions were complex, with more than one rehabilitation component, and included physical activity and management of behavioural and educational symptoms. The methodological quality was considered low. They conclude that there is “low-level” evidence for TCS interventions in reducing short-term disability and fatigue, functional activities, fatigue, pain, insomnia, and long-term psychological symptoms. The data was limited on the evaluation of the process (the satisfaction of the participants and the therapists), and no cost analysis was performed [35]. Despite the paucity of data and the low level of evidence, partly explained by methodological problems, such interventions could be an alternative method of functional and cognitive rehabilitation in MS patients, although more robust trials are needed to prove clinical effectiveness and the cost of these interventions [35, 36].

Several authors have reported that adaptation to these computer programmes is good. Amato et al. observed a benefit only in sustained attention tasks in a randomised trial conducted on 88 patients, using training with specific computerized training and nonspecific training for one hour twice a week. Regardless of the training received, the patients' perception was positive [37]. The same results in adaptation to the programme have recently been published with an application for mobile devices with memory exercises [38].

The main dilemma in this issue is that there are no programmes, computer programme or on-site, that have demonstrated superiority; then, there is no recommendation in this regard. Even more, we do not know what is the therapeutic window for cognitive rehabilitation. The efficacy of CS in neurodegenerative diseases, as MS, today is subject of controversy; maybe, it only allows a better adaptation to cognitive dysfunction.

Nevertheless, a recent Cochrane review of 2016 finds that the benefit of cognitive rehabilitation is clear in MS patients with cognitive impairment, at least in memory [39]. MS is a progressive disease, and CS can, through the neuronal plasticity of a young brain, meet both expectations: functional improvement and cognitive improvement. Other authors believe that CS does not improve cognitive performance but reduces perceived deficit in MS patients [36, 40]. Like other authors [41], we think that both goals can be achieved, as in stroke and brain damage.

This project aims to evaluate whether this type of intervention may be an alternative method in the treatment of patients with MS and cognitive impairment, given the need for more studies that prove the clinical effectiveness and cost of these interventions [30].

## 4. Conclusion

The design of our study aims to answer the question of the validity of TCS in MS patients with CI, since it has not been shown to have the same ability to maintain cortical functions as is attributed to FCS. The interest lies in the clear benefits of remote rehabilitation for patients' daily lives.

In our opinion, a feasible, cost-effective alternative that could respond to this need would be the combination of both forms of training; since in our experience, patients with CI require supervision and training in the cognitive therapy to be applied, whether face-to-face or remote.

We considered that this study could help to establish the usefulness of TCS; or in its absence, TCS with FCS helps in MS patients with CI.

## References

[1] Guijarro-Castro C., Moreno-García S., Bermejo-Pareja F., Benito-León J. (2010). Calidad de vida relacionada con la salud en Esclerosis Múltiple. *Revista Española de Esclerosis Múltiple*.

[2] Chiaravalloti N. D., DeLuca J. (2008). Cognitive impairment in multiple sclerosis. *The Lancet Neurology*.

[3] DeLuca G. C., Yates R. L., Beale H., Morrow S. A. (2015). Cognitive impairment in multiple sclerosis: clinical, radiologic and pathologic insights. *Brain Pathology*.

[4] Guijarro-Castro C., Muñoz-Pasadas M., Martínez-Alvárez A. El Symbol Digit Modality Test no discrimina pacientes de Síndrome Radiológico Aislado de controles sanos.

[5] Parmenter B. A., Weinstock-Guttman B., Garg N., Munschauer F., HB Benedict R. (2007). Screening for cognitive impairment in multiple sclerosis using the symbol digit modalities test. *Multiple Sclerosis*.

[6] Rao S. M. (1990). *A Manual for the Brief, Repeatable Battery of Neuropsychological Tests in Multiple Sclerosis*.

[7] Benedict R. H. B., Cookfair D., Gavett R. (2006). Validity of the minimal assessment of cognitive function in multiple sclerosis (MACFIMS). *Journal of the International Neuropsychological Society*.

[8] Amato M. P., Portaccio E., Goretti B. (2010). Relevance of cognitive deterioration in early relapsing-remitting MS: a 3-year follow-up study. *Multiple Sclerosis Journal*.

[9] Langdon D. W., Amato M. P., Boringa J. (2012). Recommendations for a brief international cognitive assessment for multiple sclerosis (BICAMS). *Multiple Sclerosis Journal*.

[10] Amato M. P., Langdon D., Montalban X. (2013). Treatment of cognitive impairment in multiple sclerosis: position paper. *Journal of Neurology*.

[11] Benedict R. H., Morrow S. A., Weinstock Guttman B., Cookfair D., Schretlen D. J. (2010). Cognitive reserve moderates decline in information processing speed in multiple sclerosis patients. *Journal of the International Neuropsychological Society*.

[12] Sumowski J. F., Rocca M. A., Leavitt V. M. (2014). Brain reserve and cognitive reserve protect against cognitive decline over 4.5 years in MS. *Neurology*.

[13] Rosti-Otajärvi E. M., Hämäläinen P. I. (2014). Neuropsychological rehabilitation for multiple sclerosis. *Cochrane Database of Systematic Reviews*.

[14] das Nair R., Ferguson H., Stark D. L., Lincoln N. B. (2012). Memory rehabilitation for people with multiple sclerosis. *Cochrane Database of Systematic Reviews*.

[15] das Nair R., Martin K. J., Lincoln N. B. (2016). Memory rehabilitation for people with multiple sclerosis. *Cochrane Database of Systematic Reviews*.

[16] Parisi L., Rocca M. A., Valsasina P., Panicari L., Mattioli F., Filippi M. (2014). Cognitive rehabilitation correlates with the functional connectivity of the anterior cingulate cortex in patients with multiple sclerosis. *Brain Imaging and Behavior*.

[17] Benedict R. H., Munschauer F., Linn R. (2003). Screening for multiple sclerosis cognitive impairment using a self-administered 15-item questionnaire. *Multiple Sclerosis Journal*.

[18] Arango-Lasprilla J. C., Rivera D., Rodríguez G. (2015). Symbol digit modalities test: normative data for the Latin American Spanish speaking adult population. *NeuroRehabilitation*.

[19] Folstein M. F., Folstein S. E., McHugh P. R. (1975). “Mini-mental state”. A practical method for grading the cognitive state of patients for the clinician. *Journal of Psychiatric Research*.

[20] Rilo O., Peña J., Ojeda N. (2018). Integrative group-based cognitive rehabilitation efficacy in multiple sclerosis: a randomized clinical trial. *Disability and Rehabilitation*.

[21] Tombaugh T. N. (2004). Trail making test A and B: normative data stratified by age and education. *Archives of Clinical Neuropsychology*.

[22] Gronwall D. M. A. (1977). Paced auditory serial-addition task: a measure of recovery from concussion. *Perceptual and Motor Skills*.

[23] Benedet M. J., Alejandre M. A. (1998). *TAVEC: test de aprendizaje verbal España-Complutense: manual*.

[24] Krupp L. B., LaRocca N. G., Muir-Nash J., Steinberg A. D. (1989). The fatigue severity scale. Application to patients with multiple sclerosis and systemic lupus erythematosus. *Archives of Neurology*.

[25] Hobart J., Lamping D., Fitzpatrick R., Riazi A., Thompson A. (2001). The multiple sclerosis impact scale (MSIS-29): a new patient-based outcome measure. *Brain*.

[26] Beck A. T., Ward C. H., Mendelson M., Mock J., Erbaugh J. (1961). An inventory for measuring depression. *Archives of General Psychiatry*.

[27] Fernández-Sanchez M., Sánchez J., Diago J. M., Bescos J. M. Neuropsychological rehabilitation in fibromialgia patients. Effects of an online cognitive training program.

[28] Fernández-Sanchez Barjola P., Sánchez J., Diago J. M., Bescos J. M. Evaluación de los efectos de un programa de brainfitness online sobre el rendimiento académico y neuropsicológico de un grupo de estudiantes universitarios.

[29] Sola-Valls N., Blanco Y., Sepúlveda M., Martinez-Hernandez E., Saiz A. (2015). Telemedicine for monitoring MS activity and progression. *Current Treatment Options in Neurology*.

[30] Moss-Morris R., McCrone P., Yardley L., van Kessel K., Wills G., Dennison L. (2012). A pilot randomised controlled trial of an internet-based cognitive behavioural therapy self-management programme (MS Invigor8) for multiple sclerosis fatigue. *Behaviour Research and Therapy*.

[31] Sola-Valls N., Blanco Y., Sepúlveda M. (2015). Walking function in clinical monitoring of multiple sclerosis by telemedicine. *Journal of Neurology*.

[32] Plohmann A. M., Kappos L., Ammann W. (1998). Computer assisted retraining of attentional impairments in patients with multiple sclerosis. *Journal of Neurology, Neurosurgery, & Psychiatry*.

[33] Vogt A., Kappos L., Calabrese P. (2009). Working memory training in patients with multiple sclerosis - comparison of two different training schedules. *Restorative Neurology and Neuroscience*.

[34] Mattioli F., Stampatori C., Zanotti D., Parrinello G., Capra R. (2010). Efficacy and specificity of intensive cognitive rehabilitation of attention and executive functions in multiple sclerosis. *Journal of the Neurological Sciences*.

[35] Khan F., Amatya B., Kesselring J., Galea M. (2015). Telerehabilitation for persons with multiple sclerosis. *Cochrane Database of Systematic Reviews*.

[36] Lincoln N. B., das Nair R., Bradshaw L. (2015). Cognitive rehabilitation for attention and memory in people with multiple sclerosis: study protocol for a randomised controlled trial (CRAMMS). *Trials*.

[37] Amato M., Goretti B., Viterbo R. (2014). Computer-assisted rehabilitation of attention in patients with multiple sclerosis: results of a randomized, double-blind trial. *Multiple Sclerosis Journal*.

[38] Tacchino A., Pedullà L., Bonzano L. (2015). A new App for at-home cognitive training: description and pilot testing on patients with multiple sclerosis. *JMIR mHealth and uHealth*.

[39] das Nair R., Martin K., Lincoln N. B. (2016). Memory rehabilitation for people with multiple sclerosis. *Cochrane Database of Systematic Reviews*.

[40] Mäntynen A., Rosti-Otajärvi E., Koivisto K., Lilja A., Huhtala H., Hämäläinen P. (2014). Neuropsychological rehabilitation does not improve cognitive performance but reduces perceived cognitive deficits in patients with multiple sclerosis: a randomised, controlled, multi-centre trial. *Multiple Sclerosis Journal*.

[41] Cerasa A., Tomaiuolo F., Quattrone A. (2014). Which is the goal of cognitive rehabilitation in multiple sclerosis: the improvement of cognitive performance or the perception of cognitive deficits?. *Multiple Sclerosis Journal*.

